# Size measurement and filled/unfilled detection of rice grains using backlight image processing

**DOI:** 10.3389/fpls.2023.1213486

**Published:** 2023-10-13

**Authors:** Xiao Feng, Zhiqi Wang, Zhiwei Zeng, Yuhao Zhou, Yunting Lan, Wei Zou, Hao Gong, Long Qi

**Affiliations:** ^1^ College of Engineering, South China Agricultural University, Guangzhou, Guangdong, China; ^2^ Guangdong Laboratory for Lingnan Modern Agriculture, Guangzhou, Guangdong, China; ^3^ Department of Agricultural Engineering Technology, University of Wisconsin-River Falls, River Falls, WI, United States; ^4^ R&D Center, Top-Leading Intelligent Technology Co. ltd., Guangzhou, Guangdong, China

**Keywords:** rice, breeding, physical traits, computer vision, image processing

## Abstract

Measurements of rice physical traits, such as length, width, and percentage of filled/unfilled grains, are essential steps of rice breeding. A new approach for measuring the physical traits of rice grains for breeding purposes was presented in this study, utilizing image processing techniques. Backlight photography was used to capture a grayscale image of a group of rice grains, which was then analyzed using a clustering algorithm to differentiate between filled and unfilled grains based on their grayscale values. The impact of backlight intensity on the accuracy of the method was also investigated. The results show that the proposed method has excellent accuracy and high efficiency. The mean absolute percentage error of the method was 0.24% and 1.36% in calculating the total number of grain particles and distinguishing the number of filled grains, respectively. The grain size was also measured with a little margin of error. The mean absolute percentage error of grain length measurement was 1.11%, while the measurement error of grain width was 4.03%. The method was found to be highly accurate, non-destructive, and cost-effective when compared to conventional methods, making it a promising approach for characterizing physical traits for crop breeding.

## 1 Introduction

Breeding researchers have long been exploring various ways to effectively improve rice yield, quality, and stress resistance characteristics (Zhang et al., 2007; Araus et al., 2008; Wang et al., 2015). Measurements of rice physical traits, such as length, width, and percentage of filled/unfilled grains, are essential steps of rice breeding. An accurate estimation of the physical traits will increase the effectiveness of rice breeding (Ge et al., 2021; Luo et al., 2019). However, the conventional manual measurement of these traits is time-consuming and prone to fatigue, resulting in large errors in the results. Moreover, Duan et al. (2011) suggested that it is challenging to manually distinguish filled grains from unfilled grains. Therefore, an accurate, automatic, and rapid assessment of the physical traits is necessary to effectively and efficiently improve rice yield and quality through rice breeding.

Due to the advancement of computer performance, the digital image processing method has been widely used in seed analysis due to its high efficiency (Al-Tam et al., 2013; Crowell et al., 2014; Liu et al., 2017). A Panicle Trait Phenotyping method (P-trap) was developed for high-throughput measurements of panicle architecture and seed-related traits (Faroq et al., 2013); A method to measure grain size and color from images captured with consumer-level flatbed scanners was also developed (Whan et al., 2014); A grain detection model was proposed to recognize and count grains on primary branches of rice plant by (Deng et al., 2021; Deng et al., 2022). These methods and models can detect the total grain number and size parameters with reasonable accuracy. However, they cannot distinguish filled and unfilled grains, and thus cannot count the number of filled grains.

Alternative imaging modalities, such as magnetic resonance imaging (MRI) and positron emission tomography (PET), have been explored for the investigation of plant traits, albeit their adoption remains limited in the research community (Borisjuk et al., 2012; Hubeau et al., 2015). This constrained utilization can be attributed to the predominant presence of MRI and PET scanners within the confines of hospitals and medical research centers, primarily due to the substantial capital investment and ongoing maintenance exigencies associated with these instruments. Notably, recent endeavors have employed nuclear magnetic resonance (NMR) to gauge grain weight and composition on a population scale, although this was predominantly executed on discrete, unconsolidated grains (Corol et al., 2016). Despite these incremental strides, a critical gap persists in the arsenal of techniques dedicated to the expedient and non-destructive assessment of crop yield impacts and, more specifically, grain characteristics, whilst preserving critical spatial information. In concert with controlled-environment growth facilities, the integration of advanced imaging capabilities holds the potential to furnish an unprecedented level of precision in scrutinizing the influence of environmental factors on phenotype. X-ray micro-computed tomography (μCT) emerges as a non-invasive imaging modality grounded in the principle of differential X-ray attenuation by biological specimens, offering a viable and cost-effective alternative (Rousseau et al., 2015; Tracy et al., 2017). The µCT apparatus comprises essential components, including an X-ray source, a sample rotation stage, and an X-ray detector. The attenuation of X-rays during their traversal through the specimen corresponds to the density and atomic composition of the material and is captured by the imaging detector as grayscale values. By systematically altering the orientation of either the X-ray beam or the sample, an array of projections is acquired from diverse angles, which can subsequently be reconstructed to yield an accurate three-dimensional representation or model of the object under scrutiny (Hughes et al., 2017; Dhondt et al., 2010). Originally devised for medical diagnostic purposes, recent advances in µCT technology have yielded enhancements in scan resolution, overall quality, and scan duration reduction, rendering it amenable to the investigation of intricate plant traits (Pajor et al., 2013; van der Niet et al., 2010). The capability to discern and quantify internal structures in a non-invasive and non-destructive manner, coupled with the potential for process automation, renders µCT an enticing and efficacious approach for the systematic study of plant traits. High-resolution µCT has, in fact, successfully found utility in the comprehensive analysis of various aspects, including soil properties, root architecture, developing seeds, shoot structures, emerging panicles, and leaf morphology (Staedler et al., 2013; Jhala et al., 2015). Duan et al. (2011) proposed a method of counting total grains and filled grains simultaneously, based on automatic discrimination of filled and unfilled grains by combining visible-light imaging and soft X-ray Imaging. Yu et al. (2021) adapted this method to study the structure of rice panicles. Researchers (Strange et al., 2014) used computed tomography to carry out three-dimensional imaging of wheat panicles and used image processing technology to extract grains from the three-dimensional model of the panicle for analysis. Although X-ray imaging is effective at distinguishing filled and unfilled grains, the radiation may affect the nutrients and genes of seeds, creating problems in seed breeding (Yali and Mitiku, 2022). Deep learning techniques have also been used to identify shapes and calculate the number of seeds (Uzal et al., 2018; Zhao et al., 2022).

This study proposed a phenotype acquisition method for measuring the physical traits of rice grains using visible spectrum photography and backlight image processing. The specific objectives were to: (1) propose an image processing algorithm for measuring rice grain size and detecting filled/unfilled grains; (2) investigate the effects of light intensity and dark ratio threshold on grain recognition performance; and (3) validate the accuracy of the algorithm by comparing the predicted results with experimental results.

## 2 Materials and methods

### 2.1 Image acquisition system

**Figure 1:**
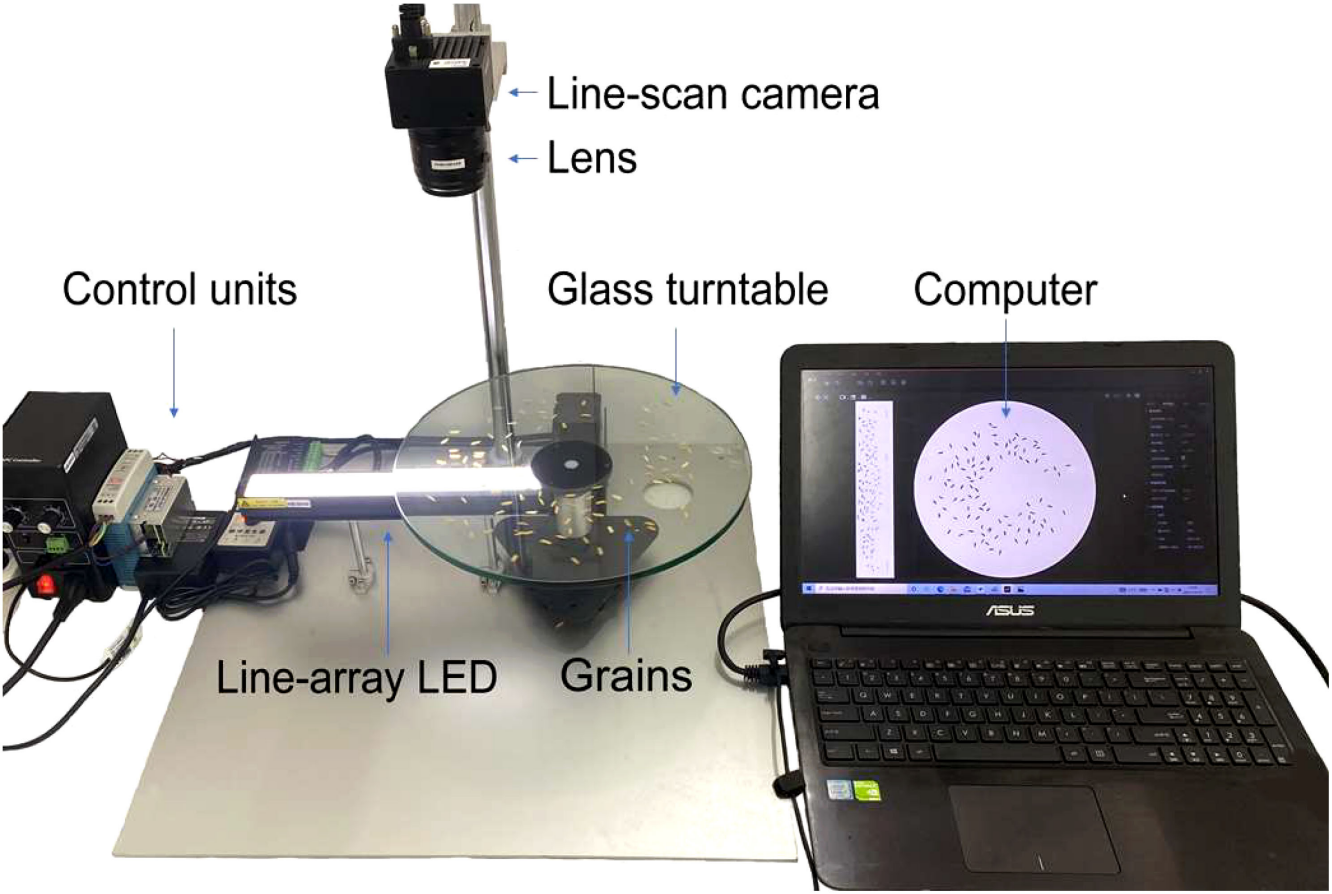
The image acquisition system.

The rice grains were placed on a glass turntable and illuminated by a white strip light underneath. A line scanning camera took continuous pictures of the grains and recorded backlight images. An image processing algorithm was developed to identify the length, width, and filled/unfilled status of each grain based on grayscale values in the backlight images. To obtain clear and high-quality images of rice grains, an image acquisition system was built as shown in **Figure 1**. The system consists of a line-scan camera MV-CL021-40GM (Hikvision Digital Technology Corporation, Hangzhou, China) with a 20 mm lens (F4.5, LS003F20-D30, CST, China), a glass turntable (Corence sensor Corporation, Ningbo, China), a white line-array LED illumination source possessing a spectral range spanning from 450 to 700 nm (Hegho Electric Corporation, Shenzhen, China), a computer and control units. The white line-array LED was placed underneath the glass turntable. The glass turntable was continuously rotated by a stepper motor with an encoder at a rate of 40 rpm. The line-scan camera (encompassing a detectable spectral range from 380 to 780 nm) captured images of the grains on the turntable at a resolution of 0.086 mm/pixel, synchronized with the encoder’s pulse. A Gigabit Ethernet cable was used to transport the backlight images from the camera to the computer for real-time processing.

### 2.2 Rice samples

Rice samples of the variety of Huahang No.57 (*Oryza sativa subsp. indica*) were obtained for testing in this study. The samples were collected on July 12, 2021, from a 10-meter-long by 4-meter-wide paddy field located at the Institute of Agricultural Sciences in Zhaoqing, Guangdong, China (23°10’9.303” N, 112°34’13.810” E). A total of 50 rice panicles at the mature stage were randomly sampled in the field. These harvested rice panicles undergo a threshing process, facilitated by a mechanized threshing apparatus, supplemented by manual intervention to facilitate the removal of extraneous grain impurities such as broken leaves, fractured stems, and residual branchlets. It is imperative to note that no further treatments, including washing or additional cleansing procedures, are administered to the rice grains. Subsequent to the completion of the threshing procedure, it is imperative that the separated grains undergo a drying process, which is executed at a controlled temperature of 40°C for a duration spanning 3 days. The actual grain sizes, the total number of grains and the number of filled grains for each panicle were measured and counted three times and the average values were used as experimental reference (in **Appendix**).

### 2.3 Experimental design

Three experiments were conducted in this study to examine the effects of parameter settings on algorithm detection results and to validate the accuracy of the results. These experiments included a backlight intensity test, a dark ratio threshold test, and a validation experiment. The first two tests focused on investigating the impact of algorithm parameters on the results while the third test aimed to validate the performance of the proposed method by comparing the results with actual values obtained from manual counting and measurements.

For the backlight intensity test, 50 grains were randomly selected from 5 panicles, with 25 being filled and the other 25 being unfilled (see **Appendix A**). The backlight intensity varied from 20 klux to 200 klux with an increment of 20 klux. The grayscale value of each grain was calculated as the summed average of the grayscale of all pixels on that grain.

For the dark ratio threshold test, 15 panicles of rice were used and the actual values of the filled and unfilled grains were measured (see **Appendix B**). Typically, each rice spike is observed to encompass a range of 130 to 290 rice grains. Consequently, when assessing the optimal dark ratio threshold, an exhaustive total of approximately 3,000 rice grains were meticulously segregated and were subsequently incorporated into the testing process. From preliminary studies, four typical dark ratio threshold values, namely 0.4, 0.6, 0.7, and 0.8, were selected to examine the effect of threshold on grain counting accuracy.

In the validation experiment, actual values of the filled and unfilled grains were measured from 30 panicles, and the sizes of 50 filled grains were randomly taken from those panicles (see **Appendix C**). The measured data were used as the reference data for comparison with the results obtained from the proposed method. The performance of the proposed method was evaluated using regression analyses.

### 2.4 Image processing

**Figure 2:**
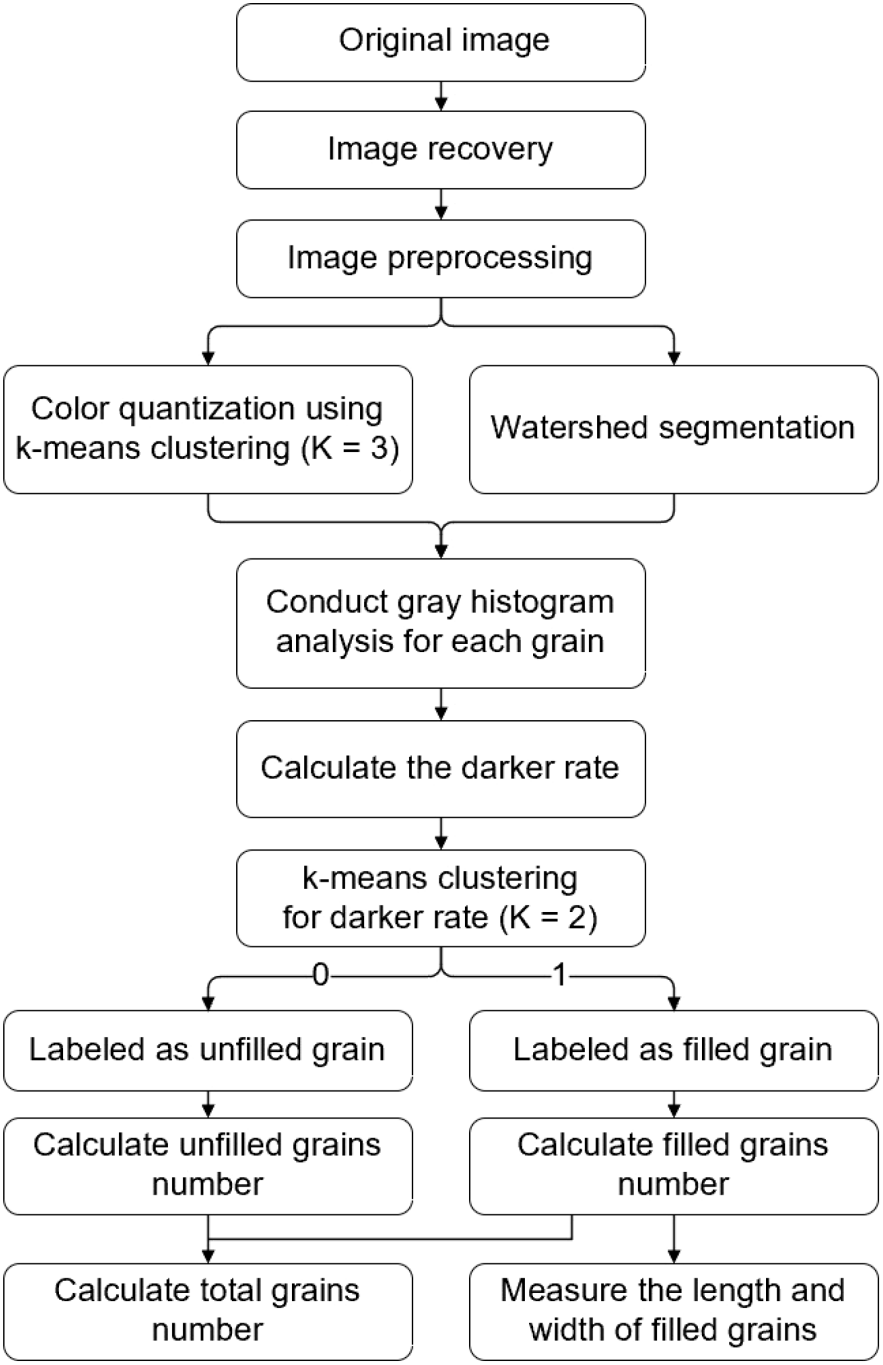
Image analysis algorithm flowchart.

An algorithm for identifying the size of each rice grain and the percentage of filled/unfilled grains was developed, and its flow chart is presented in **Figure 2**. The algorithm consists of several processes, including image remapping, image preprocessing, watershed algorithm, recognition of filled and unfilled grains, and size measurement, which are described in detail in the following sections. All programs were written using OpenCV-Python 4.5.2 (Bradski et al., 2000).

#### 2.4.1 Image remapping

**Figure 3:**
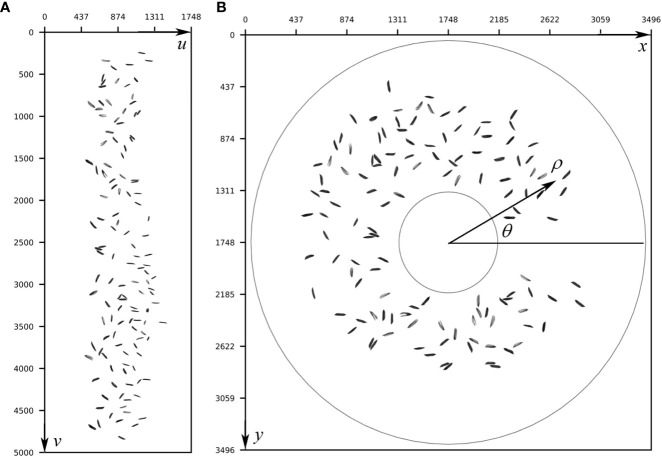
Image remapping from an original image in the Cartesian system to the restored image in the polar coordinate system; **(A)** Original image; **(B)** Remapped image.


As shown in **Figure 1**, a line-scan camera was used to take the image of grains on the turntable. Although the turntable rotated at a constant angular velocity, the linear velocities of grains with different distances to the center of the turntable were different. The linear velocities of the grains near the outer ring were greater than the grains near the inner ring of the turntable due to the greater rotating radius of the former group. As a result, the original image taken by the camera was distorted as shown in **Figure 3A**. Thus, an image remapping algorithm was proposed to solve the distortion problem. The idea of this algorithm was to convert the pixels of the original image from the Cartesian coordinate system to the polar coordinate system (Zhong and Quan, 2017) according to equations (1) to (4). The restored image is shown in **Figure 3B**.

(1) $$\rho =e^{\frac{u\log w}{w}}$$

(2) $$\theta =\frac{2\pi v}{w}$$

(3) x=w+ρcosθ

(4) y=w+ρsinθ

where (u,v) represents the coordinates of the point on the original image, w represents the total width ( u -axis) of the original image in pixel, (ρ,θ) represents the coordinates of the point on the polar coordinate system, and (x,y) is coordinates of the point on the remapped image.

#### 2.4.2 Pre-processing

Three operations including median filtering, binarization, and morphological operation were included in the pre-processing procedure. In the image, noise points may appear due to clastic grains on the glass turntable. To remove these noise points, a 5×5 median filter kernel was applied. It should be noted that the noise points that are much smaller than the grain area but cannot be filtered out would not be included in subsequent analysis. After filtering, binarization was performed. Since the backlight image had a uniform color distribution, a fixed threshold was sufficient to segment the foreground and background. Typically, the shape of each rice grain consists of an oval shell and an empty glume with a gap in between. The gap was noticeable under backlight irradiation and appeared as a small hole in the image. The proposed algorithm dilated the binary image first and then eroded it to close the small holes in the foreground object thereby removing the small black spots in the binary image.

#### 2.4.3 Watershed segmentation

**Figure 4:**
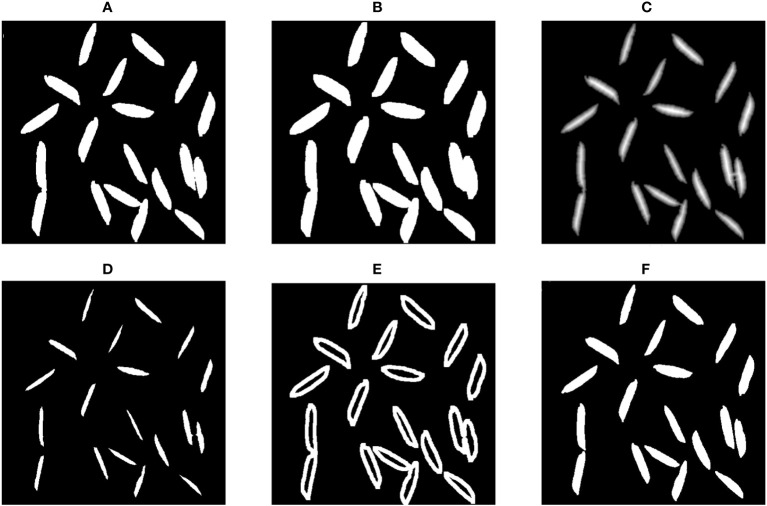
Segmentation procedures; **(A)** Original binary image; **(B)** Sure-background image; **(C)** Distance transformation image; **(D)** Sure-foreground image; **(E)** Difference; **(F)** Individual grain image.

It was observed that the grains on the glass turntable tended to be close to each other, making it necessary to use a watershed algorithm to simulate a waterflooding process and identify and divide lines between different regions. The watershed algorithm is a prominent computer vision technique employed for the purpose of image region segmentation. During the segmentation procedure, a pivotal criterion revolves around evaluating the similarity between pixels situated in close proximity within the image. This evaluation serves as a key determinant for the establishment of connections between pixels characterized by both spatial proximity and analogous grayscale values. The outcome of this process culminates in the formation of closed contours or outlines, which constitutes a hallmark feature of the watershed algorithm. In essence, this algorithm is esteemed for its capacity to accurately delineate the boundaries of objects. This article serves as an instructional resource, elucidating the application of the Watershed Algorithm in OpenCV for the task of image segmentation. The procedure of the watershed segmentation algorithm is illustrated in **Figure 4**. Initially, the algorithm performed dilation on the original binary image (**Figure 4A**) to generate the sure-background image (**Figure 4B**). Then, the distance transformation of the original binary image (**Figure 4C**) was computed, followed by thresholding to obtain the sure-foreground image (**Figure 4D**). Subsequently, the algorithm determined the unknown region (**Figure 4E**) by taking the difference between the sure-background and sure-foreground images. This unknown region was the area that requires segmentation using the watershed algorithm. Finally, the watershed algorithm segmented the unknown region to produce the individual grain images (**Figure 4F**).

#### 2.4.4 Filled/unfilled grains identification and counting

**Figure 5:**
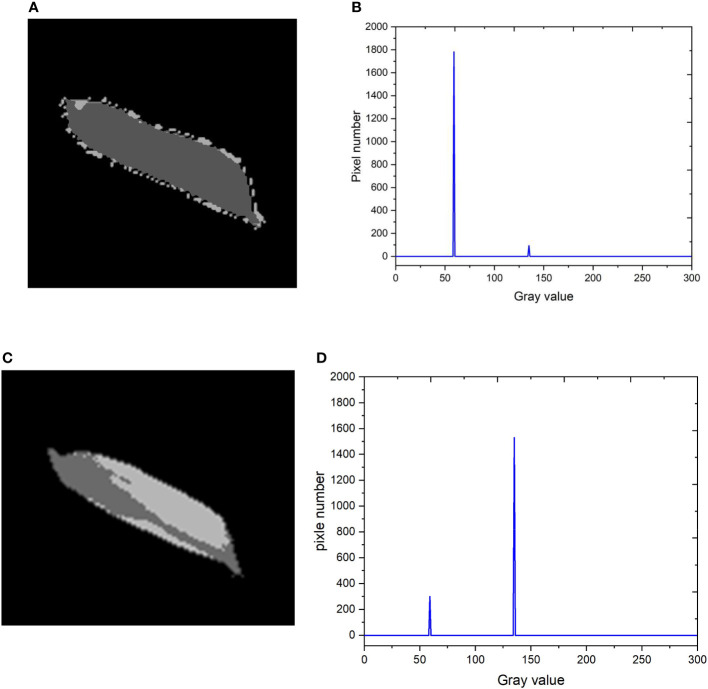
The k-means clustering results of the filled and unfilled grains; **(A)** Filled grain; **(B)** Number of light and dark pixels of the filled grains; **(C)** Unfilled grain; **(D)** Number of light and dark pixels of the unfilled grains.

The grayscale values of the backlight image were utilized to distinguish between filled and unfilled grains using the k-means clustering algorithm. By setting the number of clusters to 2, the pixel points of each grain can be separated into two groups based on their grayscale values: light pixels and dark pixels. The results of the k-means clustering algorithm for two representatives filled and unfilled grains are presented in **Figure 5**.

In order to determine whether a grain is filled or unfilled, a measurement index called “dark ratio” (or “ DR “) was introduced. This index takes into account both the number of light and dark pixels in grain by calculating the ratio of the dark pixel count to the total pixel count.


(5) $$DR=\;\frac{N_{d}}{N_{t}}$$

where *N_d_* is the number of dark pixels and *N_t_* is the total number of pixels.

**Figure 6:**
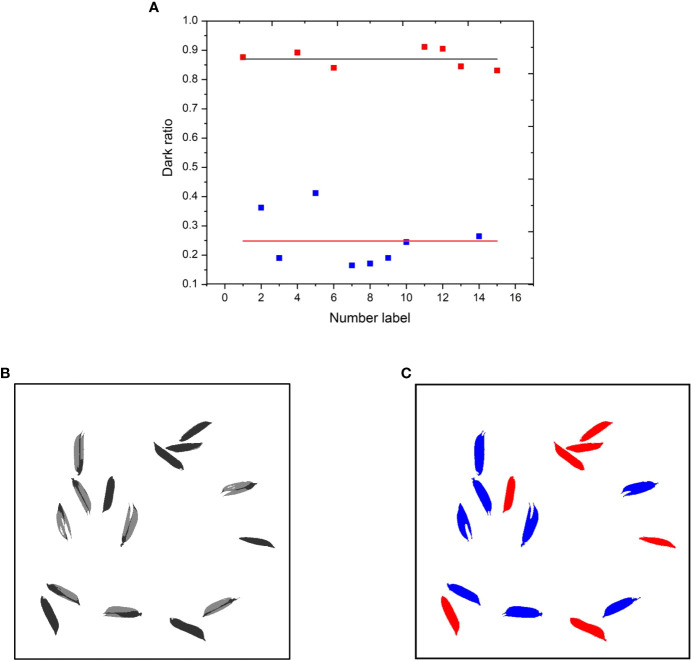
The effects of dark ratio in distinguishing filled from unfilled grains. **(A)** Dark ratio of each grain. **(B)** Original grain image. **(C)** Labeled image (Red filled, blue unfilled).

To differentiate between filled and unfilled grains, the dark ratio of each grain was computed and compared against a set threshold. If the dark ratio exceeds the threshold, the grain was classified as filled; otherwise, it was categorized as unfilled. For instance, a total of 15 grains were chosen as test subjects to illustrate the impact of the dark ratio in distinguishing filled and unfilled grains, of which 8 grains were classified as unfilled (as shown in **Figure 6**).

In **Figure 6A**, a discernible pattern emerges, wherein the dark ratios of filled grains exhibit a distribution skewed toward higher values, whereas those of unfilled grains manifest a distribution skewed toward lower values. Specifically, the average dark ratio for filled grains is quantified at 0.87, in stark contrast to the average dark ratio for unfilled grains, which stands at 0.25. This disparity between the two averages is statistically significant. Consequently, a preliminary dark ratio threshold of 0.6 has been established as a discerning criterion for distinguishing between filled and unfilled grains. It is paramount to acknowledge that threshold is susceptible to influence by an array of factors, encompassing, though not restricted to, variations in rice varieties and the presence of grain fissures, both of which can potentially engender discrepancies in the optimal dark ratio threshold. We emphasize the prospective avenues for enhancing precision by implementing adaptively optimized dark ratio thresholds, thereby paving the path towards future refinements in the accuracy of our approach.

#### 2.4.5 Size measurements

**Figure 7:**
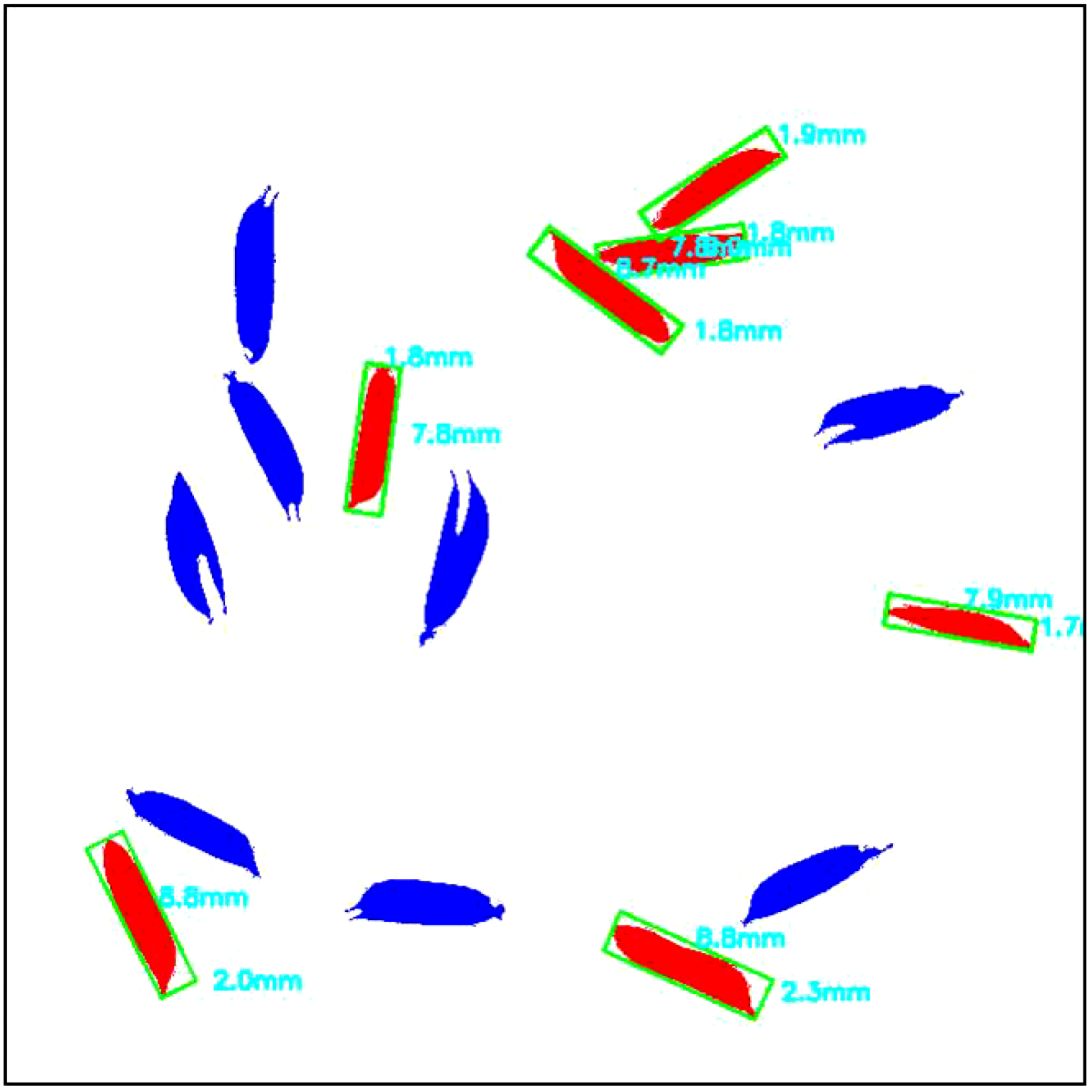
Labeled image of filled grains with sizes.

To determine the size of a grain, its length and width were measured using an optimal enclosing rectangle, as illustrated in **Figure 7**. The pixel values along the length and width of the rectangle were then converted to millimeters using the pre-set image resolution values.

### 2.5 Statistical analysis

The accuracy of the proposed method was assessed by utilizing the regression analysis technique and comparing the results with the reference data, as presented in **Appendices B, C**. The coefficient of determination (*R* ^2^), root mean square error (*RMSE*), and mean absolute percentage error (*MAPE*) were calculated as accuracy indicators of the proposed method. These metrics were computed by using the following equations:


(6) $$R^{2}=1-\frac{\sum_{j=1}^{n}{\left(m_{j}-e_{j}\right)}^{2}}{\sum_{j=1}^{n}{\left(m_{j}-\bar{m}\right)}^{2}}$$

(7) $$RMSE=\;\sqrt{\frac{\sum_{j=1}^{n}{\left(m_{j}-e_{j}\right)}^{2}}{n}}$$

(8) $$MAPE=\;\frac{1}{n}\sum_{j=1}^{n}\frac{\left|e_{j}-m_{j}\right|}{m_{j}}$$

where n is the number of samples, $e_{j}$ is the actual value, and $m_{j}$ is the predicted value by the proposed method.

## 3 Results and discussion

### 3.1 Influence of backlight intensity

**Figure 8:**
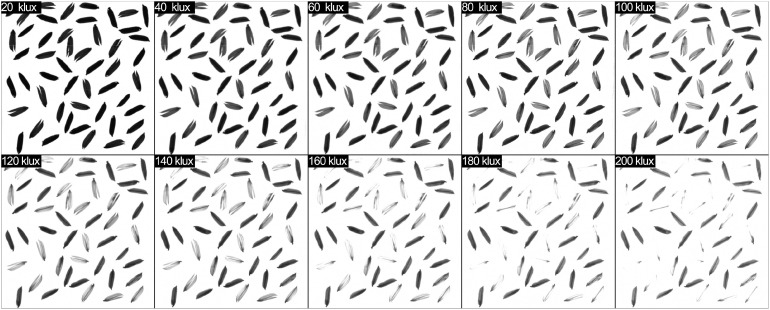
The results of different backlight intensities from 20 klux to 200 klux.

Rather than isolating and detecting color information at a specific wavelength, our approach involved the amalgamation of brightness values derived from light within the range of 380 to 780 nm, which was subsequently represented as a grayscale spectrum spanning from 0 to 255. Consequently, even in scenarios where the intensity of the backlight varied, the discrimination of filled and unfilled grains remained feasible through the computation of the cumulative brightness of transmitted light. Thus, the outcomes of our investigation remain impervious to fluctuations in the color properties associated with specific wavelengths. The impact of varying illumination intensities on the gray values of filled and unfilled grains in the image was explored in the backlight intensity test, as illustrated in **Figure 8**. The findings demonstrated that the backlight intensity had a significant effect on the quality of grain images. As the intensity of light increased, the overall brightness of the image also increased. Moreover, the visual distinctions between filled and unfilled grains became more prominent, which was beneficial for distinguishing between the two. However, when the light intensity surpassed 160 klux, the exposure increased to the point that the contour information of some unfilled grains disappeared. Conversely, when the light intensity fell below 40 klux, the visual differences between unfilled and filled grains diminished.

**Figure 9:**
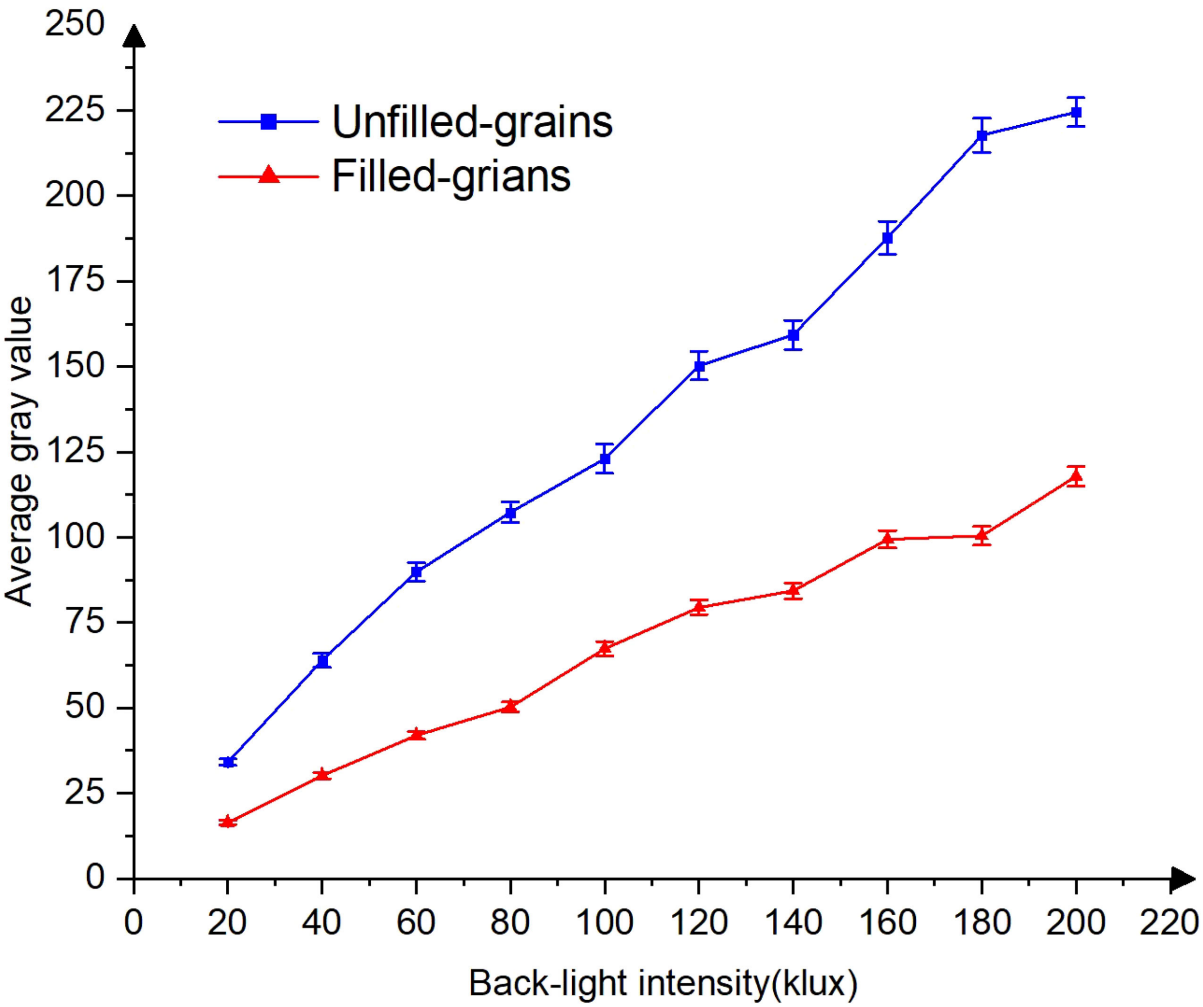
The relationship between backlight intensity and grain gray value.

To investigate the optimal backlight intensity for distinguishing between unfilled and filled grains, the average gray value of each grain was computed under various backlight intensity conditions. The average gray value is computed by summing the gray values of all pixels within each rice grain and subsequently dividing by the total number of pixels contained within that grain. The results showed a correlation between the grayscale and the intensity of backlight illumination, as presented in **Figure 9**. The average gray values of both unfilled and filled grains increased consistently when the light intensity increased from 20 to 200 klux. Nevertheless, the distinctions in gray values between filled and unfilled grains were more pronounced at 200 klux. However, Excessive illumination exceeding 140 klux may cause overexposure, potentially resulting in the miscounting of unfilled grains with thin thickness. While there was a degree of inherent variability in the absolute values of the average gray values across the various rice grains tested in this study, an overarching trend consistently emerged. This overarching trend was characterized by a discernible increase in mean gray values with rising levels of brightness.

Furthermore, the presence of cracks in covering layers of the grains exerts a notable influence on light transmission and the resulting average gray value. As a consequence, these factors contribute to an expanded range of fluctuations in the gray values associated with filled grains, engendering a visual resemblance to unfilled grains. This phenomenon becomes particularly conspicuous as the intensity of the backlighting is heightened. To illustrate this effect, it is noteworthy that under luminance conditions exceeding 180 klux, the filled grains exhibit characteristics that may be misinterpreted as unfilled grains, in contrast to those observed under illumination levels of 120 klux. In light of these considerations, we have pragmatically constrained the backlight luminance to a maximum of 120 klux. This restriction is implemented with the explicit aim of mitigating errors attributable to the presence of cracks within the seed covering layers, thereby enhancing the accuracy of our detection process.

### 3.2 Influence of dark ratio threshold

**Figure 10:**
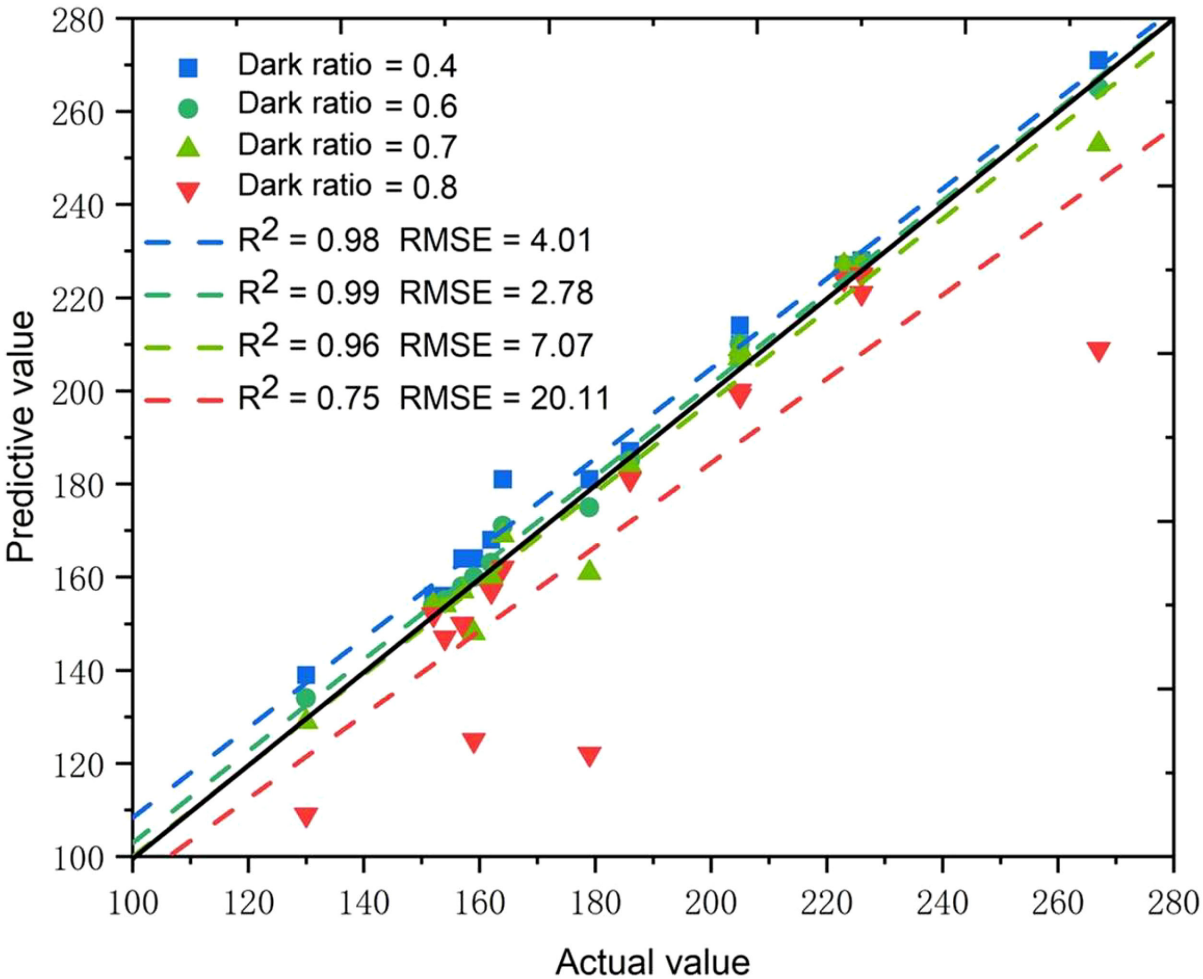
The influence of dark ratio threshold on the predicted results.

The evaluation of the dark ratio threshold involved the examination of the counting outcomes of filled grains under four distinct dark ratio thresholds, i.e., 0.4, 0.6, 0.7, and 0.8. **Figure 10** depicts the 1:1 graph of the predicted and actual values of filled grains for each threshold. The R^2^ values between the predicted and actual values were 0.98, 0.99, 0.96 and 0.75 for the dark ratio thresholds of 0.4, 0.6, 0.7, and 0.8, respectively. The corresponding RMSEs were 4.01, 2.78, 7.07, and 20.11, respectively. The system’s predicted values were higher than the actual values when the dark ratio thresholds were 0.4 or 0.6, indicating that some unfilled grains were mistakenly classified as filled grains, leading to overestimation. Conversely, the predicted values were lower than the actual values when the dark ratio thresholds were 0.7 or 0.8, indicating that some filled grains were mistakenly classified as unfilled grains, leading to underestimation. As the number of filled grains is typically higher than that of unfilled grains, using a dark ratio threshold greater than 0.6 could result in relatively large errors due to the misclassification of filled grains as unfilled grains. Therefore, a dark ratio threshold of 0.6 was chosen, as it yielded a high R^2^ of 0.99 and a low RMSE of 2.78, indicating high accuracy and consistency with the actual value.

### 3.3 Performance of the proposed method

**Figure 11:**
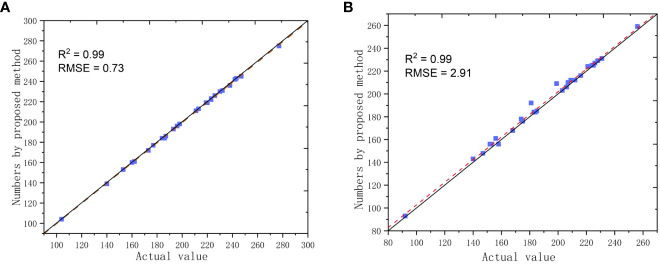
Comparisons in numbers of grains between the counted values by the proposed method ad actual values; **(A)** Total grain counts; **(B)** Filled grain counts.

The performance of the proposed method was evaluated using the reference data from the validation experiment. **Figure 11** shows the results of the total grain count and the number of filled grains counted by the proposed method and the actual values. The R^2^ and RMSE for the total grain count were 0.99 and 0.73, respectively (**Figure 11A**). The MAPE with actual values was 0.5%. These results demonstrate a high consistency between the proposed automagical counting method and actual values in terms of total grain counts. Additionally, the proposed method performed well in counting filled grains, with an R^2^ of 0.99 and an RMSE of 2.91. The MAPE with actual values was also low, at 2.43%. These findings collectively underscore the capability of our method to accurately quantify both the total number of grains and the number of actual grains, even in scenarios marked by variations in the total grain count.

It is imperative to acknowledge that the growth of rice can be susceptible to the influence of pests and diseases, which can impart alterations to both grain structure and surface color. These changes have the potential to introduce inaccuracies into our results. For instance, instances of overprediction may be ascribed to the presence of substantial black spots on the surface of certain unfilled grains, an effect induced by rice blast infestations during the growth phase. Such unfilled grains may exhibit limited light penetration, potentially leading to misidentification as filled grains. Given the significance of these variables, we underscore the promising avenue of augmenting accuracy through the integration of dynamically optimized dark ratio thresholds. However, it is essential to emphasize that our exploration of adaptive thresholding methodologies remains a work in progress. In our forthcoming research endeavors, we are committed to further enhancing the adaptability and efficacy of our algorithm, with the overarching goal of refining our analytical approach.

**Figure 12:**
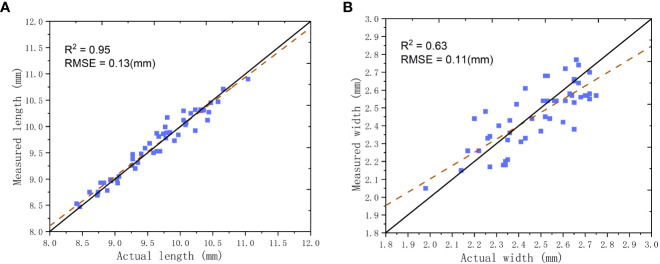
Comparisons in grain sizes between the proposed method and actual values; **(A)** Grain length; **(B)** Grain width.

The predicted and actual values of grain sizes are presented in **Figure 12**, with lengths ranging from 8.0 to 11.0 mm and widths ranging from 2.0 to 3.0 mm. The R^2^ and RMSE were 0.95 and 0.13 mm for the length and 0.63 and 0.11 mm for the width, respectively. The proposed method showed better accuracy in measuring length than width, which may be caused by the ellipsoid-shaped grain tilting slightly on the glass plane, resulting in the vertical projection width not being the maximum width of the grain. However, this effect did not affect the measurement of the length. Overall, the proposed method exhibited good performance in measuring grain characteristic parameters.

## 4 Conclusion

This study introduces an innovative method for acquiring phenotypic information about rice grains through backlight imaging. The method involves illuminating the grains from behind and capturing shadow patterns, which are then processed using computer vision algorithms to extract various physical traits. Notably, this approach offers advantages over traditional methods as it is non-destructive and radiation-free, ensuring that grains remain viable for subsequent analysis. Additionally, it enables the simultaneous measurement of multiple traits, such as total grain count, filled grain count, and grain size characteristics.

The proposed method demonstrates remarkable accuracy, achieving high consistency with actual values in terms of total grain count (R2 of 0.99 and RMSE of 0.73) and filled grain count (R2 of 0.99 and RMSE of 2.91). It also excels in length (R2 of 0.95 and RMSE of 0.13 mm) and width (R2 of 0.63 and RMSE of 0.11 mm) measurements. Overall, this innovative approach promises to significantly enhance the efficiency and accuracy of evaluating rice physical traits while remaining cost-effective. Consequently, it has the potential to revolutionize rice breeding, leading to improvements in rice production and quality control.

## Data availability statement

The original contributions presented in the study are included in the article/supplementary material. Further inquiries can be directed to the corresponding authors.

## Author contributions

XF and ZW worked out the technical details and drafted the manuscript with support from ZZ. HG and LQ were involved in planning and supervised the work. YZ, YL and WZ processed the experimental data, performed the analysis, and designed the figures. All authors contributed to the article and approved the submitted version.
