# Supplementary material for: Size measurement and filled/unfilled detection of rice grains using backlight image processing
Source: Front Plant Sci. 2023 Oct 13;14:1213486. doi: 10.3389/fpls.2023.1213486 (PMC10613065; doi:10.3389/fpls.2023.1213486)
Supplement: Supplementary file 1 [file Table_1.docx]

1. **Appendix A**

Table 1. Panicles used in backlight intensity test (Actual values)

| Panicle | Total number of grains | Filled grain number | Unfilled grain number |
| --- | --- | --- | --- |
| 1 | 159 | 148 | 11 |
| 2 | 245 | 234 | 11 |
| 3 | 324 | 311 | 13 |
| 4 | 115 | 105 | 10 |
| 5 | 98 | 88 | 10 |

1. **Appendix B**

Table 2. Reference grain number used in dark ratio threshold test (Actual values)

| Panicle | Total number of grains | Filled grain number | Unfilled grain number |
| --- | --- | --- | --- |
| 6 | 136 | 130 | 6 |
| 7 | 190 | 179 | 11 |
| 8 | 160 | 154 | 6 |
| 9 | 175 | 159 | 16 |
| 10 | 291 | 267 | 24 |
| 11 | 194 | 162 | 32 |
| 12 | 187 | 157 | 30 |
| 13 | 220 | 205 | 15 |
| 14 | 209 | 164 | 45 |
| 15 | 241 | 226 | 15 |
| 16 | 237 | 226 | 11 |
| 17 | 227 | 205 | 22 |
| 18 | 159 | 152 | 7 |
| 19 | 233 | 223 | 10 |
| 20 | 199 | 186 | 13 |

1. **Appendix C**

Table 3. Actual values of the grain number in validation test

| Panicle | Total grain number | Filled grain number | Unfilled grain number |
| --- | --- | --- | --- |
| 21 | 139 | 127 | 12 |
| 22 | 186 | 175 | 11 |
| 23 | 153 | 147 | 6 |
| 24 | 172 | 158 | 14 |
| 25 | 275 | 256 | 19 |
| 26 | 184 | 152 | 32 |
| 27 | 184 | 156 | 28 |
| 28 | 222 | 207 | 15 |
| 29 | 219 | 174 | 45 |
| 30 | 243 | 228 | 15 |
| 31 | 242 | 231 | 11 |
| 32 | 231 | 209 | 22 |
| 33 | 161 | 153 | 8 |
| 34 | 236 | 226 | 10 |
| 35 | 198 | 184 | 14 |
| 36 | 219 | 206 | 13 |
| 37 | 223 | 212 | 11 |
| 38 | 226 | 216 | 10 |
| 39 | 226 | 216 | 10 |
| 40 | 245 | 224 | 21 |
| 41 | 193 | 183 | 10 |
| 42 | 213 | 181 | 32 |
| 43 | 243 | 221 | 22 |
| 44 | 104 | 92 | 12 |
| 45 | 243 | 199 | 44 |
| 46 | 177 | 168 | 9 |
| 47 | 160 | 140 | 20 |
| 48 | 230 | 225 | 5 |
| 49 | 196 | 185 | 11 |
| 50 | 211 | 203 | 8 |

Table 4. Grain number measured by proposed method in validation test

| Panicle | Total grain number | Filled grain number | Unfilled grain number |
| --- | --- | --- | --- |
| 21 | 140 | 132 | 8 |
| 22 | 187 | 175 | 12 |
| 23 | 153 | 148 | 5 |
| 24 | 173 | 156 | 17 |
| 25 | 277 | 259 | 18 |
| 26 | 184 | 155 | 29 |
| 27 | 186 | 160 | 26 |
| 28 | 223 | 212 | 11 |
| 29 | 220 | 180 | 40 |
| 30 | 243 | 231 | 12 |
| 31 | 242 | 231 | 11 |
| 32 | 232 | 212 | 20 |
| 33 | 162 | 156 | 6 |
| 34 | 238 | 228 | 10 |
| 35 | 198 | 184 | 14 |
| 36 | 219 | 206 | 13 |
| 37 | 223 | 212 | 11 |
| 38 | 226 | 216 | 10 |
| 39 | 226 | 218 | 8 |
| 40 | 247 | 225 | 22 |
| 41 | 193 | 185 | 8 |
| 42 | 213 | 189 | 24 |
| 43 | 243 | 224 | 19 |
| 44 | 104 | 94 | 10 |
| 45 | 243 | 208 | 35 |
| 46 | 177 | 170 | 7 |
| 47 | 160 | 143 | 17 |
| 48 | 230 | 225 | 5 |
| 49 | 196 | 185 | 11 |
| 50 | 211 | 204 | 7 |

Table 5. The sizes of the grains in validation test

| Grain | Length  (Actual values) | Width  (Actual values) | Length measured by proposed method | Width measured by proposed method |
| --- | --- | --- | --- | --- |
| 1 | 10.09 | 2.61 | 10.06 | 2.61 |
| 2 | 9.26 | 2.66 | 9.19 | 2.64 |
| 3 | 9.8 | 2.72 | 9.97 | 2.73 |
| 4 | 10.44 | 2.52 | 10.30 | 2.45 |
| 5 | 9.53 | 2.53 | 9.58 | 2.59 |
| 6 | 9.77 | 2.39 | 9.82 | 2.39 |
| 7 | 9.74 | 2.51 | 9.75 | 2.40 |
| 8 | 9.97 | 2.68 | 9.88 | 2.62 |
| 9 | 10.58 | 2.26 | 10.49 | 2.33 |
| 10 | 9.64 | 2.71 | 9.80 | 2.51 |
| 11 | 11.04 | 2.52 | 10.93 | 2.54 |
| 12 | 10.08 | 2.61 | 10.04 | 2.60 |
| 13 | 9.78 | 2.75 | 9.80 | 2.71 |
| 14 | 9.27 | 2.67 | 9.36 | 2.65 |
| 15 | 10.16 | 2.63 | 10.20 | 2.62 |
| 16 | 9.67 | 2.72 | 9.74 | 2.71 |
| 17 | 9.69 | 2.53 | 9.66 | 2.55 |
| 18 | 9.79 | 2.65 | 9.81 | 2.64 |
| 19 | 9.06 | 2.5 | 9.04 | 2.51 |
| 20 | 8.73 | 2.25 | 8.70 | 2.24 |
| 21 | 8.88 | 2.35 | 8.84 | 2.25 |
| 22 | 10.05 | 2.43 | 10.14 | 2.41 |
| 23 | 9.27 | 1.98 | 9.31 | 2.02 |
| 24 | 8.46 | 2.27 | 8.47 | 2.28 |
| 25 | 9.64 | 2.65 | 9.56 | 2.62 |
| 26 | 9.31 | 2.46 | 9.29 | 2.44 |
| 27 | 10.36 | 2.56 | 10.30 | 2.54 |
| 28 | 8.74 | 2.41 | 8.68 | 2.35 |
| 29 | 8.83 | 2.34 | 8.86 | 2.35 |
| 30 | 10.23 | 2.72 | 10.26 | 2.74 |
| 31 | 9.35 | 2.14 | 9.32 | 2.13 |
| 32 | 10.66 | 2.31 | 10.66 | 2.36 |
| 33 | 10.23 | 2.2 | 10.11 | 2.30 |
| 34 | 8.41 | 2.17 | 8.44 | 2.14 |
| 35 | 8.78 | 2.22 | 8.87 | 2.15 |
| 36 | 8.93 | 2.27 | 8.94 | 2.38 |
| 37 | 8.61 | 2.34 | 8.66 | 2.30 |
| 38 | 10.42 | 2.54 | 10.25 | 2.54 |
| 39 | 9.4 | 2.64 | 9.42 | 2.61 |
| 40 | 9.03 | 2.33 | 9.00 | 2.34 |
| 41 | 8.94 | 2.36 | 8.94 | 2.35 |
| 42 | 9.04 | 2.35 | 9.01 | 2.30 |
| 43 | 9.59 | 2.67 | 9.55 | 2.66 |
| 44 | 10.47 | 2.65 | 10.45 | 2.56 |
| 45 | 10.32 | 2.43 | 10.29 | 2.49 |
| 46 | 10.05 | 2.6 | 10.07 | 2.52 |
| 47 | 9.84 | 2.57 | 9.85 | 2.54 |
| 48 | 9.91 | 2.36 | 9.80 | 2.40 |
| 49 | 9.46 | 2.65 | 9.53 | 2.67 |
| 50 | 10.31 | 2.7 | 10.27 | 2.68 |
